# WHEN OLDER ADULTS GOT TRAUMATIC INJURY: EFFECTS OF CASE MANAGEMENT

**DOI:** 10.1093/geroni/igad104.2065

**Published:** 2023-12-21

**Authors:** Hsueh-Fen Kao, Sarah Jimenez

**Affiliations:** The University of Texas at El Paso, El Paso, Texas, United States; University of Texas at El Paso, El Paso, Texas, United States

## Abstract

Case management can improve patient outcomes from the acute to rehabilitation phase after traumatic injury. However, a lack of evidence on effects of case management in older patients with traumatic injury makes it difficult to translate research findings into practice. The study was to examine the effects of a case management intervention on illness perception, coping strategies used, and quality of life in older patients with traumatic injury across a 9-month span after hospital discharge. A 4-wave longitudinal, experimental design was used in older patients with traumatic injury hospitalized at a regional hospital in Taiwan. Subjects were randomly assigned to case management (experimental) or usual care (control) group. The intervention started during hospitalization and ended with a phone follow-up two weeks post discharge. Illness perception and/or coping strategies used along with health-related quality of life were measured at baseline, 3, 6 and 9 months after discharge. Generalized estimating equations were used for analysis. Findings showed significant differences of illness perceptions at 3 and 6 months as well as coping strategies used at 6 and 9 months after discharge between the two groups. However, no significant difference on quality of life over time between the two groups was found. While case management appears to help older patients with traumatic injury decrease illness perception and better cope with their injury, it does not significantly improve their quality of life nine months after discharge. It is recommended that healthcare professionals develop a comprehensive long-term case management program for older patients with traumatic injury.

